# Primary thyroid fibrosarcoma in a 32-year-old female: case report and literature review

**DOI:** 10.1093/jscr/rjae298

**Published:** 2024-05-14

**Authors:** Fouad Jaber, Mark Rahal, Amira Shikh Alkassabin, Hanin Hamza, Salim Haddad, Mohamad Shbat, Hussain Chaban, Zein Basha, Sultaneh Haddad

**Affiliations:** Department of Surgery, Faculty of Medicine, Damascus University, Damascus 30621, Syrian Arab Republic; Albasel Hospital, Department of Surgery, Homs, Syrian Arab Republic; Department of Surgery, Faculty of Medicine, Damascus University, Damascus 30621, Syrian Arab Republic; Department of Pediatrics, Faculty of Medicine, Aleppo University, Aleppo 12212, Syrian Arab Republic; Department of Surgery, Faculty of Medicine, Damascus University, Damascus 30621, Syrian Arab Republic; Department of Surgery, Assad University Hospital, Damascus, Syrian Arab Republic; Department of Surgery, Assad University Hospital, Damascus, Syrian Arab Republic; Department of Surgery, Assad University Hospital, Damascus, Syrian Arab Republic; Department of Pediatrics, Faculty of Medicine, Aleppo University, Aleppo 12212, Syrian Arab Republic; Department of Scientific Research, Stemosis for Scientific Research, Damascus, Syrian Arab Republic

**Keywords:** fibrosarcoma, thyroid, tumor

## Abstract

Thyroid fibrosarcomas represent a rare subset of tumors with exceedingly limited documented cases in the medical literature. This study delineates an unusual occurrence involving a 32-year-old female presenting with symptoms including neck pain, dysphagia, and dyspnea. Notably, the patient experienced symptom recurrence 3 months postthyroidectomy, accompanied by aggressive tumor growth. Despite the considerable size of the tumor and its infiltration into critical anatomical structures, a complex surgical intervention was executed with successful outcomes. The study underscores the imperative for further exploration into the efficacy of proposed therapeutic modalities tailored for managing this neoplasm. Moreover, it emphasizes the necessity for considering the histological classification of fibrosarcoma within the differential diagnoses spectrum for thyroid tumors.

## Introduction

Fibrosarcoma is a malignant tumor originating from mesenchymal cells within fibrous connective tissue, primarily comprising aggressive fibroblasts embedded in a collagen matrix [1]. Thyroid fibrosarcomas represent an exceedingly rare subset of tumors, with minimal documented cases reported in the medical literature [2]. Despite extensive research, the etiological factors contributing to the development of this disease remain elusive. Although it may arise from previously damaged skin areas (such as burns, trauma, chemical exposure, or radiotherapy), it typically manifests de novo from normal tissue [1].

Clinically, thyroid fibrosarcoma commonly presents as a painless enlarging mass, often associated with respiratory and swallowing difficulties attributed to compression of neck structures [2]. Advances in diagnostic methodologies, including ultrasound (US), computed tomography (CT), magnetic resonance imaging (MRI), thyroid scintigraphy, and fine-needle aspiration biopsy (FNAB), have significantly improved diagnostic accuracy [2].

Treatment approaches typically involve surgical excision, with or without adjunctive chemotherapy and brachytherapy, or palliative care in advanced cases [2]. However, the risk of local recurrence postresection is considerable, estimated at 50%, and each recurrence tends to exacerbate tumor grade and prognosis, thereby increasing the likelihood of metastasis [3]. The prognosis remains dismal, with a notably shortened life expectancy [1].

## Case presentation

**Figure 1:**
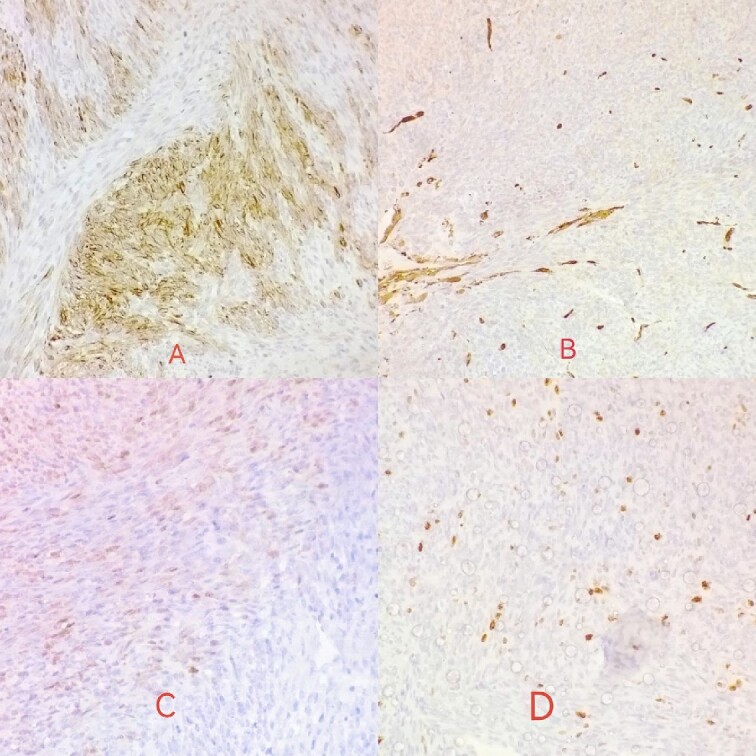
Desmin is focally positive in tumor cells (A); actin is negative in tumor cells (B); S100 reveals mild focal nuclear positivity (C); ki67 proliferative index <10% (D).

This study presents an unusual case involving a 32-year-old non-smoker woman with a medical history of cesarean section and laparoscopy for adhesiolysis, who presented with neck pain, dysphagia, and dyspnea. Clinical examination revealed a solid, tender, and mobile mass extending into the right and central regions of the neck. Laboratory findings indicated concomitant hypothyroidism. US examination showed the presence of a substantial tissue lesion, exhibiting dimensions of ~4 by 8 cm, which manifests as a heterogeneous hypoechoic anomaly, positioned along the midline with a discernible right-sided prevalence. Its impact is notable in displacing the right cervical vessels laterally and elevating the right thyroid lobe. FNAB yielded the presence of atypia of undetermined significance (Bethesda 3). To evaluate the stage of the disease, neck US and chest and neck CT were done without noticing any metastases. Complete thyroidectomy with mass excision was done without complications. The surgeon confirmed that the tumor occupied the thyroid gland, demonstrating a neoplastic proliferation originating from within its confines, which was also evident on the US. No other masses were observed in the neck or around the thyroid gland. Histopathological examination reported the product of total thyroidectomy with isthmectomy that measured 50 x 35 x 20 mm and weighed 28 g, associated with multiple separate segments of lobulated homogenous white mass, which, combined, measured 120 x 20 mm and weighed 144 g. Microscopic examination showed spindle cell neoplasm. Tumor cells were diffusely positive for vimentin but focally positive for desmin and S100 (Fig. 1) and negative for CK and TTF1 with ki67 proliferative index <10%. The final interpretation of the histopathology study was consistent with fibrosarcoma. However, 3 months postthyroidectomy, the patient’s symptoms persisted, and the mass exhibited aggressive growth.

**Figure 2:**
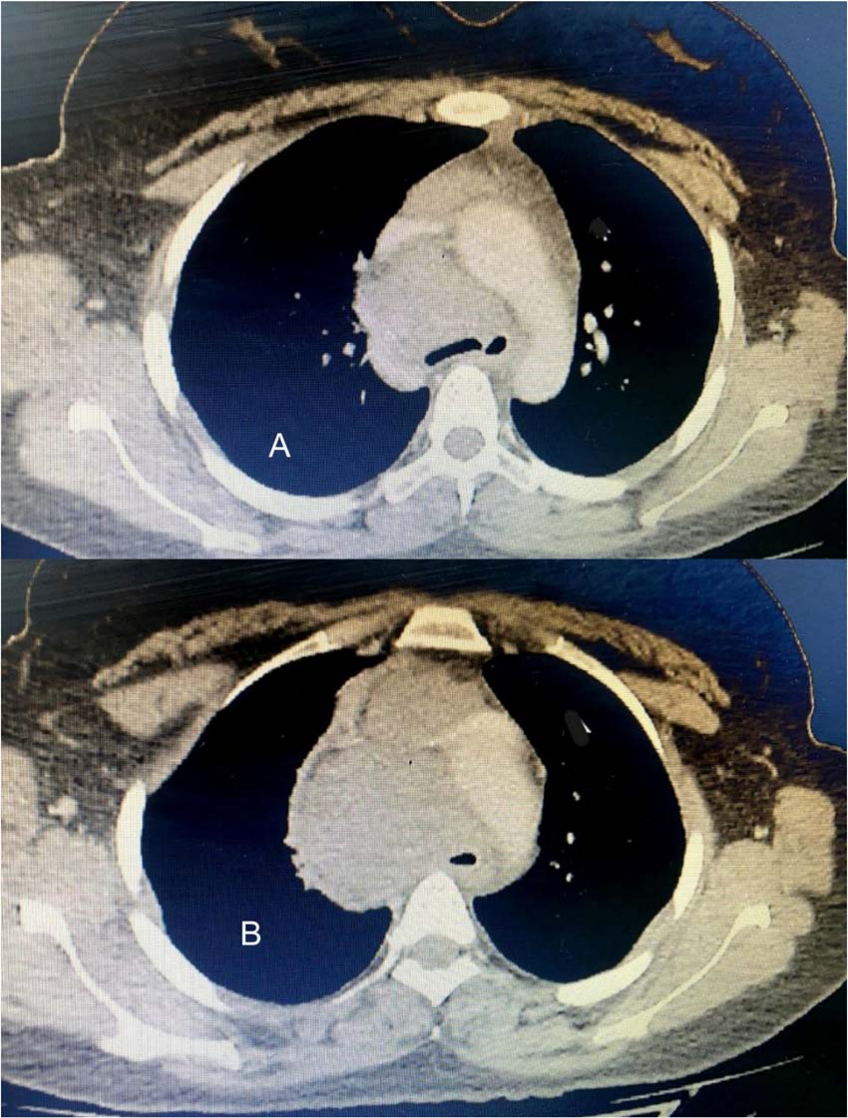
(A) The tumor extends up to the tracheal carina and compresses on the aortic arch; (B) a tumor in the mediastinum closes the trachea almost completely, deflects it toward the left, and infiltrates the aortic arch and the vena cava.

**Figure 3:**
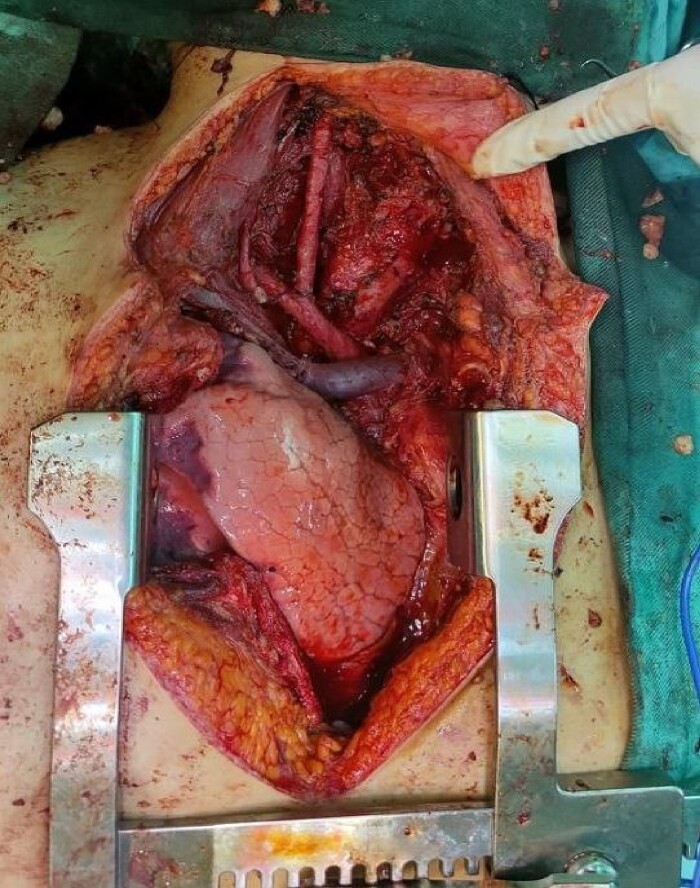
Wide Kocher incision, vital organs after removing the tumor.

Six chemotherapy courses were instituted, comprising doxorubicin (75 mg/m^2^) and ifosfamide (7.5 mg/m^2^) every 3 weeks. Unfortunately, they failed to yield improvement; instead, the mass continued to enlarge. CT-guided injection of the neck and chest revealed a formidable, heterogeneous, hypodense mass with calcifications, measuring 10 × 9 × 15 cm, predominantly occupying the thyroid space and infiltrating adjacent structures, including the right common carotid artery and innominate vein (Fig. 2). A second surgical intervention was imperative, involving a wide Kocher incision (Fig. 3) under general anesthesia. Intraoperatively, the mass was identified as infiltrating the right common carotid artery and innominate vein, necessitating meticulous dissection and shear deployment. The mass extended to the trachea and carina tracheae, prompting careful removal following exposure and isolation of the right and left phrenic nerves.

The wound was closed in layers without any complications. Two units of blood were transfused during the surgical procedure.

The patient developed symptoms and signs of hypocalcemia after surgery, complaining of numbness and tingling around the mouth and fingers. Clinical examination revealed positive Trousseau’s sign and Chvostek’s sign. Treatment was administered by intravenous infusion of calcium gluconate in 5% dextrose solution.

**Figure 4:**
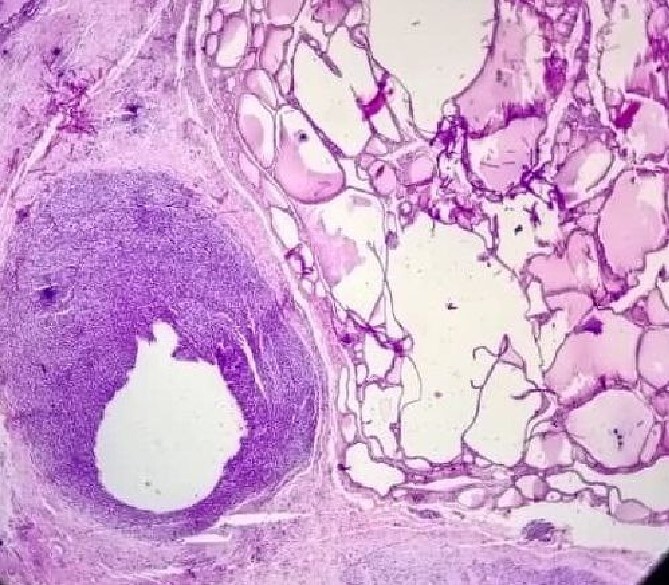
The tumor invading of the thyroid tissue.

The postoperative pathological study shown in (Fig. 4) clearly shows the tumor invading the thyroid tissue.

The patient continued chemotherapy, but 3 months after surgery, there was a metastasis in the lung and suprasternal area.

## Discussion

Papillary carcinoma stands as the most prevalent form of thyroid malignancy, followed by follicular carcinoma in terms of frequency. Less commonly encountered types within the spectrum of epithelial-origin thyroid malignancies include medullary carcinoma and Hurthle cell carcinoma. Anaplastic thyroid cancer, while relatively rare, exhibits an aggressive nature, distinguishing it as a variant with heightened malignancy potential among thyroid cancers [4]. Thyroid sarcomas are considered exceedingly uncommon neoplasms, constituting <0.2% of all documented thyroid malignancies [5].

Within this rare subset, fibrosarcoma emerges as a distinct entity, comprising ~9.2% of primary thyroid sarcomas (PTS) as revealed by a comprehensive literature review encompassing all 142 cases reported globally over 24 years from 1990 to 2014 [6].

Fibrosarcomas are defined as malignant neoplasms composed of fibroblasts that may have varying amounts of collagen production and a ‘herringbone’ architecture [7].

Fibrosarcomas can be subdivided into 2 types: infantile or congenital fibrosarcomas and adult-type fibrosarcomas [8].

Unlike the infantile variant, which is characterized by the World Health Organization as an infrequently metastasizing intermediate malignancy, fibrosarcoma observed in adults is categorized as a markedly aggressive neoplasm [9].

Adult-type fibrosarcoma commonly manifests in the lower extremities, notably in the thighs, knees, arms, and trunk, owing to their collagen-rich connective tissue composition. In contrast, occurrences of fibrosarcoma in the retroperitoneum, mediastinum, head, or neck regions are less frequently diagnosed [10].

Thyroid sarcomas typically manifest as painless, sizable goiters exhibiting varying growth rates, some rapidly expanding, while others demonstrate a gradual, progressive increase in size. These growth patterns might lead to accompanying symptoms like dyspnea, coughing, and dysphagia, arising from compression or infiltration of nearby structures [6].

Although the US, CT, and MRI can provide valuable insights into the dimensions, compression, or infiltration of surrounding structures, they cannot offer specific results for diagnosis. However, only histopathological and immunohistochemical analyses can lead to a conclusive diagnosis [2].

In our case, the tumor is a monomorphic spindle cell proliferation showing no more than moderate nuclear pleomorphism; fascicular and herringbone architecture.

The immunohistochemical profile does not show any specific differentiation: only vimentin (the Pan mesenchymal marker) shows prominent positivity as mentioned in the main text.

Therefore, a diagnosis of exclusion was established based on H&E staining coupled with immune staining findings.

Although no definitive guidelines exist for treating sarcomas, surgery remains the primary therapeutic approach, recognized for its effectiveness. However, controversies persist concerning the utility of chemotherapy and radiotherapy. Typically, these additional treatments are considered in cases where the tumor removal is incomplete or when infiltration into adjacent structures occurs. Despite their application, the actual benefits of chemotherapy and radiotherapy remain subject to debate within the medical community, highlighting the ongoing lack of consensus regarding their efficacy [2].

In alignment with findings from a systematic review on PTS [6], the onset of the tumor was not discerned during the third decade of life, as observed in our specific case; rather, its occurrence was observed across a spectrum of ages extending from the latter part of the fourth decade to the eighth decade [1, 2, 11–15].

The expansion of the tumor to a substantial magnitude, akin to our specific case, went unnoticed, except in a singular instance where the dimensions reached 13 cm [13].

The surgical intervention was notably circumvented in a case involving the encroachment upon vital organs [1], a pattern we also encountered. Nevertheless, in our case, a successful surgical procedure was accomplished.

Although the precise pathogenesis of the tumor remains elusive, it has been observed that two cases are correlated with radiation exposure, either as a consequence of the therapeutic interventions [14], or arising from the aftermath of war [12]. One of the cases was associated with AIDS [15].

Although unanimity exists among all cases regarding pathological autopsy as the definitive diagnostic modality, a conspicuous absence of a universally therapeutic approach was noted [1, 2, 11–15].

Chemotherapeutic interventions proved inefficacious in our specific case, with recurrence manifesting promptly and voluminously. The optimal course of treatment for this tumor remains to be determined.

## Conclusion

Fibrosarcoma rarely manifests in the thyroid gland, with only a limited number of documented cases in the literature over the preceding decades. Despite the potential utility of imaging modalities, histopathology remains the definitive gold standard for diagnosis. There is a compelling need for further investigations into the efficacy of proposed therapeutic modalities for this neoplasm. The histological classification of fibrosarcoma must be considered within the spectrum of differential diagnoses for thyroid tumors, irrespective of the patient’s youth and the tumor’s rapid growth characteristics.

## References

[1] Dabelić N , Mateša N, Jukić T, et al. Primary fibrosarcoma of the thyroid gland: case report. Acta Clin Croat 2016;55:172–5. 10.20471/acc.2016.55.01.24. 27333734

[2] Tucciarone M , Heredia-Llinas C, Lowy-Benoliel A, et al. Giant thyroid fibrosarcoma – a case report. Iran J Otorhinolaryngol 2020;32:109–12. 10.22038/ijorl.2019.39741.2310. 32219077 PMC7085927

[3] Stylianidi MC , Haeberle L, Schott M, et al. Primary thyroid gland myxofibrosarcoma: a case report and review of the literature. Surg Case Rep 2022;8:139. 10.1186/s40792-022-01496-5. 35876910 PMC9314473

[4] Melak T , Mathewos B, Enawgaw B, Damtie D. Prevalence and types of thyroid malignancies among thyroid enlarged patients in Gondar, Northwest Ethiopia: a three-year institution based retrospective study. BMC Cancer 2014;14:899. 10.1186/1471-2407-14-899. 25465399 PMC4289368

[5] Buła G , Waler J, Niemiec A, et al. Unusual malignant thyroid tumors--a clinical study of 20 cases. Acta Chir Belg 2008;108:702–7. 10.1080/00015458.2008.11680320. 19241922

[6] Surov A , Gottschling S, Wienke A, et al. Primary thyroid sarcoma: a systematic review. Anticancer Res 2015;35:5185–91. 26408676

[7] Davis DD , Shah SJ, Kane SM. Fibrosarcoma. *StatPearls [Internet]*. 2023 Nov 12. Treasure Island (FL): StatPearls Publishing, 2023. 32809594

[8] Augsburger D , Nelson PJ, Kalinski T, et al. Current diagnostics and treatment of fibrosarcoma–perspectives for future therapeutic targets and strategies. Oncotarget 2017;8:104638–53. 10.18632/oncotarget.20136. 29262667 PMC5732833

[9] Fletcher CDM, Unni KK, Mertens F, eds. Pathology and genetics of tumours of soft tissue and bone. Vol. 4. IARC, 2002.

[10] Cormier JN , Pollock RE. Soft tissue sarcomas. CA Cancer J Clin 2004;54:94–109. 10.3322/canjclin.54.2.94. 15061599

[11] Darouassi Y , Attifi H, Zalagh M, et al. Myxofibrosarcoma of the thyroid gland. Eur Ann Otorhinolaryngol Head Neck Dis 2014;131:385–7. 10.1016/j.anorl.2013.09.004. 24702999

[12] Sichel JY , Wygoda M, Dano I, et al. Fibrosarcoma of the thyroid in a man exposed to fallout from the Chornobyl accident. Ann Otol Rhinol Laryngol 1996;105:832–4. 10.1177/000348949610501012. 8865782

[13] Shin WY , Aftalion B, Hotchkiss E, et al. Ultrastructure of a primary fibrosarcoma of the human thyroid gland. Cancer 1979;44:584–91. 10.1002/1097-0142(197908)44:2<584::aid-cncr2820440227>3.0.co;2-s. 476570

[14] Tamada A , Makimoto K, Tasaka Y, et al. Radiation-induced fibrosarcoma of the thyroid. J Laryngol Otol 1984;98:1063–6. 10.1017/s0022215100148029. 6491491

[15] Ally N , Kumar D, Wodajo FM. Synchronous fibrosarcoma and medullary thyroid cancer in a man with AIDS. Am J Clin Oncol 2006;29:532–3. 10.1097/01.coc.0000177913.82468.ba. 17023794

